# Benchmarking Monte-Carlo dose calculation for MLC CyberKnife treatments

**DOI:** 10.1186/s13014-019-1370-5

**Published:** 2019-09-18

**Authors:** P.-H. Mackeprang, D. Vuong, W. Volken, D. Henzen, D. Schmidhalter, M. Malthaner, S. Mueller, D. Frei, W. Kilby, D. M. Aebersold, M. K. Fix, P. Manser

**Affiliations:** 1 Division of Medical Radiation Physics and Department of Radiation Oncology, Inselspital, Bern University Hospital, and University of Bern, Bern, Switzerland; 2 Accuray Incorporated, Sunnyvale, CA USA

**Keywords:** CyberKnife, Monte Carlo, Benchmarking, Dose calculation, TPS, QA

## Abstract

**Background:**

Vendor-independent Monte Carlo (MC) dose calculation (IDC) for patient-specific quality assurance of multi-leaf collimator (MLC) based CyberKnife treatments is used to benchmark and validate the commercial MC dose calculation engine for MLC based treatments built into the CyberKnife treatment planning system (Precision MC).

**Methods:**

The benchmark included dose profiles in water in 15 mm depth and depth dose curves of rectangular MLC shaped fields ranging from 7.6 mm × 7.7 mm to 115.0 mm  × 100.1 mm, which were compared between IDC, Precision MC and measurements in terms of dose difference and distance to agreement. Dose distributions of three phantom cases and seven clinical lung cases were calculated using both IDC and Precision MC. The lung PTVs ranged from 14 cm^3^ to 93 cm^3^. Quantitative comparison of these dose distributions was performed using dose-volume parameters and 3D gamma analysis with 2% global dose difference and 1 mm distance criteria and a global 10% dose threshold. Time to calculate dose distributions was recorded and efficiency was assessed.

**Results:**

Absolute dose profiles in 15 mm depth in water showed agreement between Precision MC and IDC within 3.1% or 1 mm. Depth dose curves agreed within 2.3% / 1 mm. For the phantom and clinical lung cases, mean PTV doses differed from − 1.0 to + 2.3% between IDC and Precision MC and gamma passing rates were > =98.1% for all multiple beam treatment plans. For the lung cases, lung V20 agreed within ±1.5%. Calculation times ranged from 2.2 min (for 39 cm^3^ PTV at 1.0 × 1.0 × 2.5 mm^3^ native CT resolution) to 8.1 min (93 cm^3^ at 1.1 × 1.1 × 1.0 mm^3^), at 2% uncertainty for Precision MC for the 7 examined lung cases and 4–6 h for IDC, which, however, is not optimized for efficiency but used as a gold standard for accuracy.

**Conclusions:**

Both accuracy and efficiency of Precision MC in the context of MLC based planning for the CyberKnife M6 system were benchmarked against MC based IDC framework. Precision MC is used in clinical practice at our institute.

## Introduction

The implementation of stereotactic radiosurgery (SRS), stereotactic radiotherapy (SRT), and stereotactic body radiotherapy (SBRT) has always been associated with high demands on dosimetry for the accurate and safe delivery of the corresponding treatments [1–3].

The Monte Carlo (MC) method plays a key role exploiting the statistical nature of the photons’ and their secondary particles’ interactions. It is generally considered as golden standard for the fundamental investigation of particle interaction processes, relevant for both measurement and calculation of dose distributions. As an example, the MC method is effectively used to determine measurement based correction factors, which are crucial especially in small field dosimetry [4–6].

One of the drawbacks of MC based solutions is the fact that MC methods are very computationally expensive. Depending on parameters like the intended statistical uncertainty, size of the problem (i.e. voxel size, calculation volume, etc.), it might be necessary to spend hours for the computation of MC based dose distributions, affecting the clinical transferability of MC methods. However, our group developed several strategies to overcome this limitation without making unacceptable compromises in terms of accuracy [7–10]. With respect to stereotactic treatments, we recently developed a vendor independent dose calculation (IDC) framework for the calculation of dose distributions for the CyberKnife® M6 radiosurgery system (Accuray Inc., Sunnyvale, CA) equipped with the InCise™ multileaf collimator (MLC) [11]. The IDC framework has been validated against measurements and showed only small differences in the order of 2% dose difference or 2 mm distance to agreement between calculated and measured dose distributions. As a consequence, the IDC framework serves not only as a highly accurate method for dose calculation, but is also useful for verification purposes. It may be possible to reduce the clinical workload for patient specific quality assurance (QA) by replacing cumbersome measurements with IDC.

More recently, a new MC based dose calculation method was released by Accuray as part of the TPS for stereotactic treatment planning purposes using the CyberKnife M6 equipped with the InCise MLC. While MC based dose calculation has been available for CyberKnife treatments using Cone and Iris collimators for several years [12, 13] and is well validated [14–16], no such validation exists for the newly introduced MC algorithm for MLC treatments. In the following, we refer to this implementation as Precision MC. The aim of this work is to benchmark this commercially available dose calculation algorithm by comparing Precision MC calculated dose distributions and resulting dose volume histogram (DVH) based parameters with the corresponding results using the IDC framework. For this purpose, the accuracy, efficiency, and usability of Precision MC are examined in academic and clinically motivated situations.

## Material and methods

### Precision MC

In our institute, the Precision MC was installed in November 2017 and after commissioning and validation it was clinically introduced for patient treatments [17]. For this purpose, during the commissioning phase, the Precision MC source model parameters were adjusted such that calculated output factors (OF), dose profiles and tissue-phantom ratio (TPR) curves match to the corresponding measurements.

The Precision MC dose calculation algorithm is described by Ma et al. [12] with implementation details information given by Heidorn et al. [18] and only a brief summary is provided here. A measurement based virtual source model is used to sample photons generated in the treatment head. A single virtual source, consistent with the linac target, is included, and its properties (energy spectrum, source point distribution, and direction distribution) are commissioned by comparison of measured and calculated TPR, dose profile, and OF. A CT based patient model is derived by determining a mass density and material type at each voxel. Mass density is assigned from a user defined Hounsfield unit (HU) to mass density calibration curve. Material type is assigned based on mass density to be either air, soft tissue, or bone. Material type is only used for photon interaction calculations. During dose calculation, each photon sampled from the source model is transported through the CT based patient model. Photon interactions are calculated using material type and mass density. At each interaction site a pre-simulated particle track (containing the details of all subsequent interactions and energy loss) is selected at the appropriate energy, aligned with the photon track and overlaid onto the patient model. Energy deposition is calculated using the pre-generated steps in this track, scaled by local mass density. These pre-simulated tracks are generated in a uniform water phantom and provided as a data library with Precision MC. This track repeating method, together with other variance reduction methods such as Russian Roulette, photon splitting, and forced photon interaction enables an efficient MC-based handling of the transport of charged particles and leads to reduced simulation times when compared to full MC simulations. Table 1 provides details about the CT conversion and interaction handling in Precision MC. In this study, Precision version 1.1.1 was used for all calculations. This was running on a Dell T7910 with 2 × 2.40 GHz CPU and 64 GB RAM.

**Table 1:** CT to voxel mass density and material type conversion for photons, electrons and positrons in both Precision MC and IDC

CT data conversion	Precision MC		IDC	
	Voxel mass density	Voxel material type	Voxel mass density	Voxel material type
Particle type				
Photons (primary and secondary)	User defined CT-HU to mass density calibration curve, identical to IDC	Mass density to material type. Material is either air, soft tissue, or bone	User defined CT-HU to mass density calibration curve, identical to Precision MC	CT material calibration curve based on Vanderstraeten et al 2017
Electrons and Positrons	As above	Water	As above	As above

### IDC

The IDC framework has been described in [11] and is used in this work for benchmarking the Precision MC algorithm. For this purpose, the IDC was commissioned based on measured OFs, dose profiles and depth dose data from the same CyberKnife M6 system. It is important to note that the IDC framework was not intended to work as a clinical tool for treatment planning but was developed in order to serve either as a patient specific QA tool or to serve as a benchmark tool (as in this study). Also for the IDC, EGSnrc served as a basis but in contrast to Precision MC, the IDC framework simulates all particles within a specific material according to the methods described in [11]. By this means, there is no pre-simulation applied resulting in computation times, which are expected to be longer than those of Precision MC. More details about the IDC implemented method is given in Table 1. All MC transport parameters remained identical to [11] with a global photon cut energy of 10 keV and 700 keV for electrons. Bremsstrahlung cross-sections were Bethe-Heitler and photon cross sectional data were read from the XCOM library.

### Benchmarking

In order to benchmark the accuracy and general performance of Precision MC, cases were either created artificially or originated from clinical routine (i.e. were clinical cases previously treated with CyberKnife using fixed size cone or Iris collimators and now re-planned using MLC and Precision MC). Starting with simplified situations (academic cases), complexity is increased to treatment plans applied to phantoms (phantom cases) and finally to clinical cases as detailed below.

The benchmark consists of the comparison of the calculated dose distribution of Precision MC and the corresponding dose distribution as either calculated by IDC or measured on the CyberKnife M6 system.

#### Academic cases

**Fig. 1:**
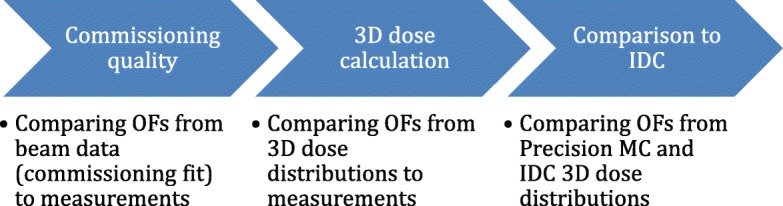
Schematic diagram describing the benchmarking of Precision MC in three steps. Blue arrows show the examined concepts and bullet points give the example of benchmarking OFs. Apart from OFs, step 2 and 3 were performed for dose profiles in 15 mm depth and depth dose curves

The basic setup for the benchmarking study is similar to the setup used to commission the IDC system [11], but now compared to matching calculations from Precision MC. A homogeneous water tank with a size of 201 × 201 × 200 mm^3^ is used. Within this water tank OFs, dose profiles, and depth dose curves are measured by a PTW 60019 microDiamond (PTW-Freiburg GmbH, Freiburg, Germany) detector or calculated using IDC and Precision MC. For this purpose, rectangular fields were shaped by the Incise MLC and different field sizes ranging from 7.6 mm × 7.7 mm to 115.0 mm × 100.1 mm were investigated. These dose quantities have already been used for the commissioning of both the IDC [11] and Precision MC models. It should be noted, however, that this measured dataset is similar, but not identical. First of all a different detector was used for the commissioning: Specifically, during commissioning, the dose profiles, the TPR’s at all field sizes, and OF’s at the smallest field size were measured using a SFD diode detector (IBA Dosimetry GmbH, Schwarzenbruck, Germany) instead of the microDiamond. No corrections were applied to the OF measured using either detector. Moreover, TPR data were used for the commissioning of the Precision MC model, while PDD data was used for IDC commissioning as well as the benchmarking study. Therefore, the simple academic test described here allows not only to validate the dose calculation accuracy but also the quality of the commissioning itself. As a first step, commissioning quality is assessed. Based upon this, data from 3D dose distributions is compared to measurements. Only then can data from Precision MC be benchmarked against IDC. These steps are illustrated in Fig. 1 for the example of OFs.

The first step is assessing the quality of the commissioning by comparing OFs from Precision MC beam data tables (originating from the commissioning process itself and thus only available in Precision MC) to measurements. Building upon this, OFs, dose profiles at 15 mm depth and depth dose curves are extracted from 3D dose distributions and compared to measurements. The third step then compares OFs, dose profiles and depth dose curves from 3D dose distributions between IDC and Precision MC. While IDC commissioning results have been presented previously for SFD measurements [11], this work updates results with microDiamond measurements. All dose profiles, depth dose curves and OFs are measured and calculated with a source-to-surface distance (SSD) of 785 mm. Calculation in the homogeneous water tank is performed using a voxel size of 1 × 1 × 1 mm^3^.

#### Phantom cases

For benchmarking purposes of a dose calculation algorithm, it is important to investigate the impact of inhomogeneities [19]. In radiation therapy, this is typically accomplished using phantoms such as lung or pelvis phantoms. In this study, one lung and two prostate treatment plans were generated on a thorax and a male pelvis phantom, respectively (CIRS IMRT thorax / CIRS IMRT pelvic 3D phantom, CIRS, Inc., Norfolk, VA). Besides the thorax phantom plan, all treatment plans were generated using sequential optimization and Table 2 summarizes treatment plan characteristics of these cases.

**Table 2:** Treatment plan characteristics

Case	Tumor site	PTV size [cc]	CT voxel size [mm]	Averaged voxel size (used for analysis) [mm]	Beams	MLC segments	Prescribed dose [Gy]	Prescription isodose [%]
1	Lung phantom	9	0.711 × 0.711 × 0.625	1.42 × 1.42 × 1.25	1	1	46.08	80
2	Prostate phantom	47	0.633 × 0.633 × 0.625	1.27 × 1.27 × 1.25	47	151	33.13	80
3	Prostate phantom	47	0.633 × 0.633 × 0.625	1.27 × 1.27 × 1.25	26	82	32.58	80
4	Lung	83	0.977 × 0.977 × 1.250	1.95 × 1.95 × 2.50	21	56	50.5	76
5	Lung	93	1.089 × 1.089 × 1.0	2.18 × 2.18 × 2.00	23	58	50.0	75
6	Lung	39	0.977 × 0.977 × 2.50	1.95 × 1.95 × 2.5	24	52	50.0	74
7	Lung	19	1.172 × 1.172 × 1.50	2.34 × 2.34 × 1.5	19	37	50.0	75
8	Lung	22	1.086 × 1.086 × 1.250	2.17 × 2.17 × 2.50	22	53	60.0	76
9	Lung	21	0.859 × 0.859 × 1.0	1.72 × 1.72 × 2.00	21	31	50.0	73
10	Lung	24	0.824 × 0.824 × 1.50	1.65 × 1.65 × 1.50	24	48	50.76	79

The thorax phantom is outfitted with a 20 mm diameter soft tissue equivalent spherical volume representing a tumor. A simplified treatment plan referred to as case 1 is generated, containing a single MLC shaped beam conformed to the tumor volume insert. For this case a single dose of 46 Gy to the 80% isodose line was prescribed and corresponding dose distributions were calculated by Precision MC, IDC and, additionally for illustration purposes, the finite size pencil beam (FSPB) algorithm implemented in Precision (the lateral kernel scaling option was not used).

For the first prostate treatment plan (case 2), a dose of 33.13 Gy was prescribed to the 80% isodose line. Sequential optimization was used to create a treatment plan with 47 beams and 151 segments. To avoid calculation uncertainties in small fields, a subset of this treatment plan was created (case 3) by manually erasing small MLC apertures (so called “perimeter shapes”, which can be enabled during optimization to fill possible underdosage at the edge of the PTV). This subset retained 26 beams and 84 segments. The number of MU of the remaining beams was unaltered, resulting in a prescribed dose of 32.58 Gy.

#### Clinical cases

In order to investigate the difference between Precision MC and IDC, clinical cases were used and dose distributions were recalculated by both algorithms. By this means, dose distributions of 7 lung treatment plans were calculated using Precision MC as well as IDC. A dose prescription of 50 Gy by 5 fractions or 60 Gy by 3 fractions to the 74–79% isodose lines was used. Depending on the case, this results in 19–24 beams and 31–58 segments.

### Calculation details

Dose distributions calculated in Precision MC targeted a statistical uncertainty of 1% for phantom and 2% for lung cases after smoothing, and were calculated at the native CT voxel resolution (i.e. as shown in the CT voxel size column of Table 2). Dose distributions from Precision MC are smoothed with a Gaussian kernel. IDC dose calculations were also performed with the calculation volume and voxel size equaling the 3D CT data set. IDC dose distributions were calculated to mean statistical uncertainties of < 1.9% in voxels receiving 50% of the respective dose maximum for all cases. For analysis, the resolution of each dose distribution (Both Precision MC and IDC) was reduced by a factor of two along any axis with a voxel edge length of < 1.5 mm. Analysis was then performed with the voxel size given in Table 2 under “averaged voxel size”.

### Analysis

Resulting dose distributions were benchmarked by analyzing dose differences, DVHs and by performing 3D gamma evaluation [20]. In all DVHs, the dose axis represents dose relative to the Precision MC dose maximum (100%) and volume relate to the volume of each contoured structure. Gamma evaluation was performed with 2% dose difference (global, relative to Precision MC dose maximum), 1 mm distance to agreement criteria and a global 10% dose threshold and IDC calculated dose was set as reference for gamma evaluation. Furthermore, mean dose to the PTV and relative lung volume receiving 20 Gy or more (lung V20) were compared between Precision MC and IDC. To analyze efficiency, calculation times for the 7 clinical lung cases were recorded in Precision MC.

## Results

### Academic cases

The commissioning process resulted in optimized virtual source model parameters for Precision MC, which are 6.3 MeV for the kinetic energy and 2.5 mm FWHM Gaussian intensity distribution of the primary electron beam. Within Precision MC, calculated OFs are saved in corresponding tables of the commissioning workspace. The comparison between these initial OFs from the commissioning and the corresponding measured OFs reveals an agreement within ±0.2%, indicating good quality of the Precision MC commissioning. This also reflects the best agreement, which can be realized in any of the upcoming experiments, since it actually describes the quality of the commissioning itself.

Investigating OFs calculated by manually setting up a treatment plan with a single beam incident on a water phantom at 785 mm SSD for a full 3D water tank, and extracting them from calculated dose distributions shows an agreement within ±1.7% between both IDC and measurement (Table 3, column 4) and between Precision MC and IDC (Table 3, column 5), except for the smallest field size. For the smallest field size, the disagreement of the OF by Precision MC with measurements is − 1.9%, while IDC determines the OF to be 2.5% lower than Precision MC. Table 3 details the comparison between OFs calculated using Precision MC, IDC, and measurement.

**Table 3:** Output Factors. Column 2 shows commissioning quality as the Precision MC commissioning process fit OFs to measured

Field Size [mm]	(Commissioning fit - measured) / measured [%]	(Precision MC - measured) / measured [%]	(IDC – measured) / measured [%]	(Precision MC - IDC) / IDC [%]
7.6 × 7.7	0.1	− 1.9	−4.3	2.5
15.4 × 15.4	− 0.1	− 1.4	− 1.1	− 0.3
23.0 × 23.1	−0.1	−1.0	− 0.5	−0.5
30.8 × 30.8	−0.1	−1.2	− 0.1	− 1.0
38.4 × 38.4	0.1	−1.0	0.0	−1.0
46.2 × 46.2	0.0	−1.2	−0.3	− 0.9
53.8 × 53.9	0.0	−2.0	−0.2	−1.7
69.2 × 69.3	0.1	−1.2	−0.5	− 0.6
84.6 × 84.7	0.2	0.5	−0.2	0.7
100.0 × 100.1	0.1	− 0.8	−0.2	− 0.7
115.0 × 100.1	0.0	0.2	−0.3	0.4

**Fig. 2:**
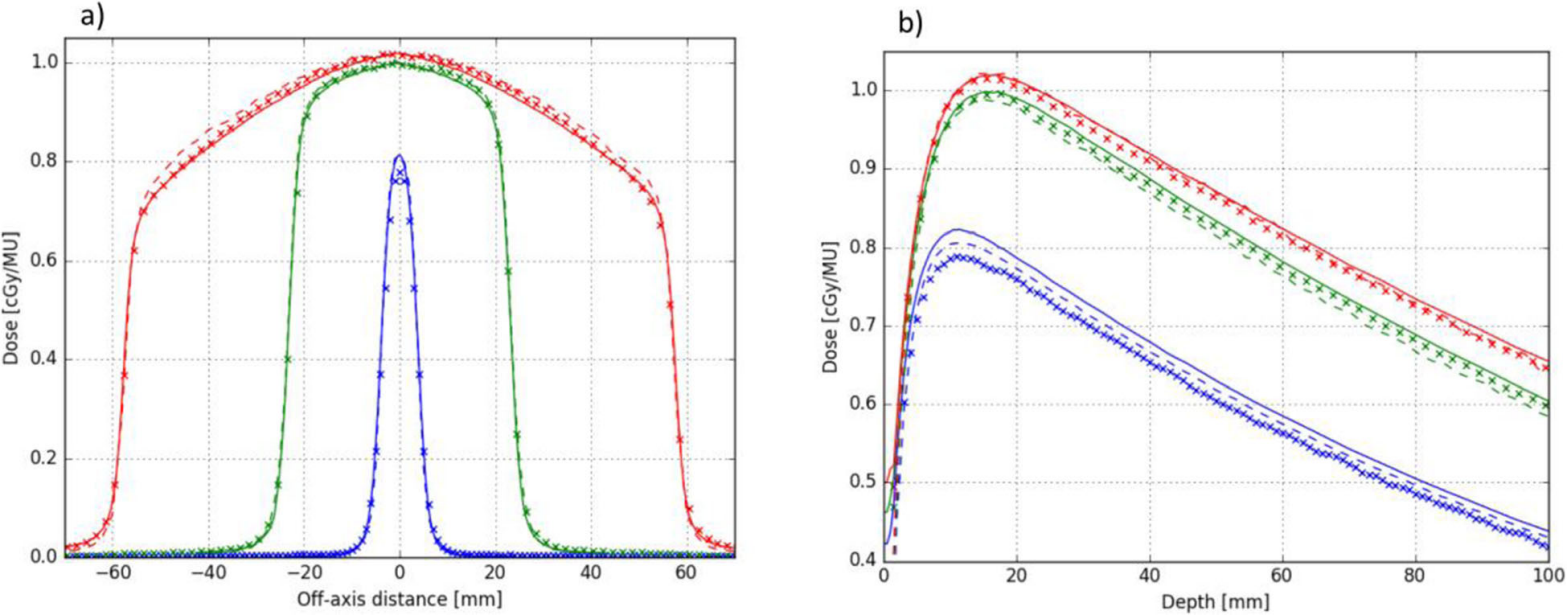
Dose profiles (**a**) along MLC leaf travel direction and depth dose curves (**b**) for microDiamond measurements (solid), Precision MC (dashed) and IDC (symbols) for 7.6 mm × 7.7 mm (blue), 46.2 mm × 46.2 mm (green) and 115.0 mm × 100.1 mm (red) rectangular MLC fields in water

Figure 2 shows dose profiles in 15 mm depth along leaf travel direction (Fig. 2a) and depth dose curves (Fig. 2b) in a homogeneous water tank for microDiamond measurements, Precision MC and IDC. For better clarity, the plots only display selected results from the smallest (7.6 mm × 7.7 mm), an intermediate (46.2 mm × 46.2 mm) and the largest (115.0 mm × 100.1 mm) rectangular MLC fields. Dose profiles at a depth of 15 mm in water show an agreement within ±3.1% or 1 mm between Precision MC and IDC for all 11 analyzed field sizes with select sizes shown in Fig. 2a. The largest discrepancy was found in the dose profile shoulder region of the two largest of all assessed field sizes, where Precision MC calculates higher doses than IDC. Between Precision MC and measurements, dose profiles agreed within ±2.2% or 1 mm.

In Fig. 2b, depth dose curves are shown. Depth dose curves agree within ±2.3% or 1 mm over all 11 assessed field sizes between Precision MC and IDC, and the largest difference is observed for the smallest MLC field size (7.6 mm × 7.7 mm), where the IDC framework calculates lower doses than measurement and Precision MC. Between Precision MC and measurement, depth dose curves also agree within 2.3% or 1 mm for all assessed field sizes.

### Phantom cases

**Fig. 3:**
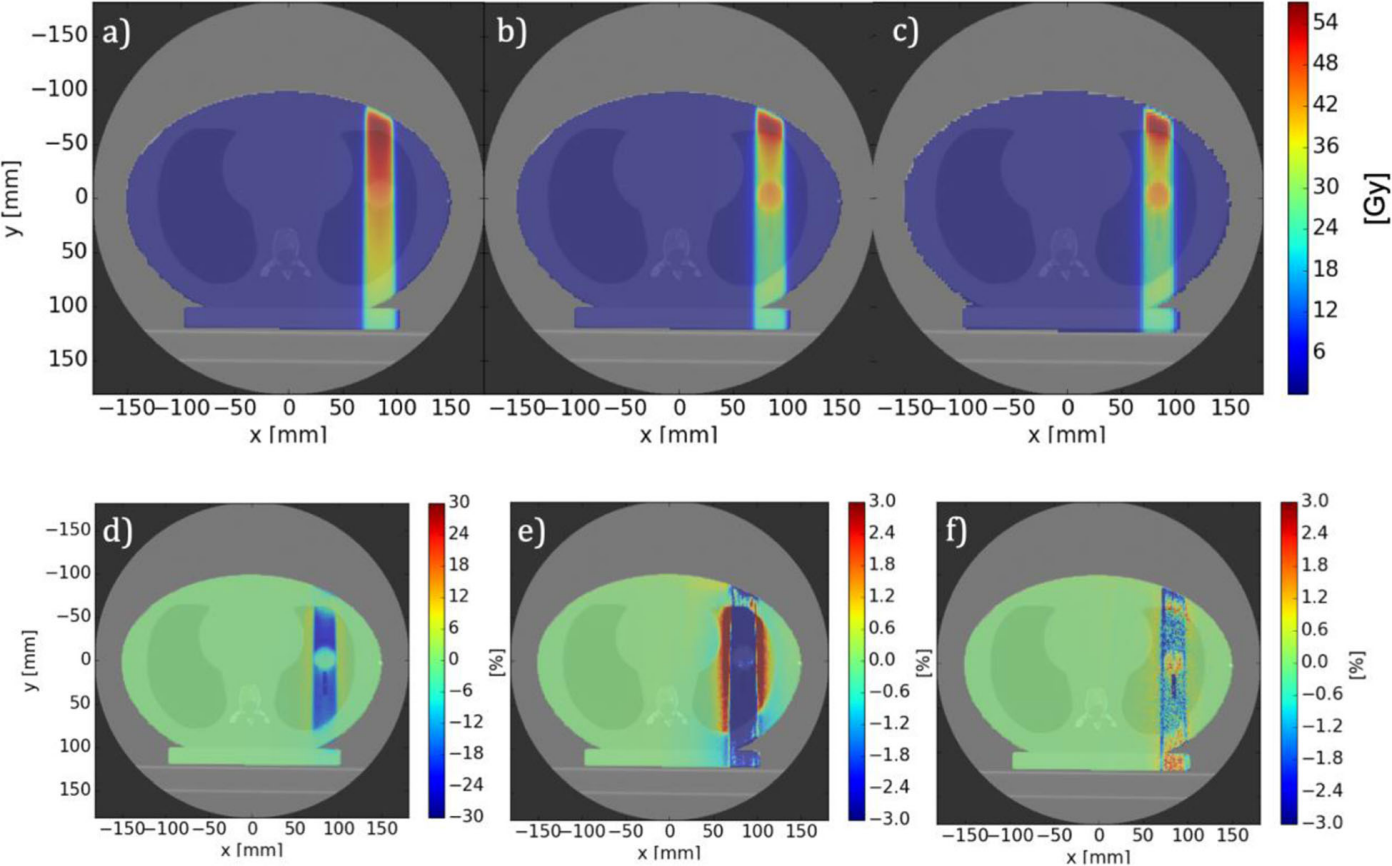
Transversal dose distributions of the single (approximately 20 mm diameter) beam lung case as calculated by (**a**) the Precision FSPB algorithm (**b**) Precision MC (**c**) IDC, respectively and resulting dose differences for (**d**) and (**e**) (IDC – Precision FSPB) / Dmax (Precision FSPB) and (**f**) (IDC – Precision MC) / Dmax (Precision MC). Note the different scales for (**d**, **e** and **f**)

The first phantom case considered demonstrates the impact of the MC based dose calculation method, when compared with Precision FSPB. As can be seen from Fig. 3, for a single beam incident on a lung case, the FSPB algorithm results in a 9.3% overestimation of the mean PTV dose when compared with IDC or Precision MC. The comparison between IDC and Precision FSPB also reveals small dose differences at the phantom boundaries to air and large differences (> 25%) in lung equivalent material inside the phantom (Fig. 3d).

**Fig. 4:**
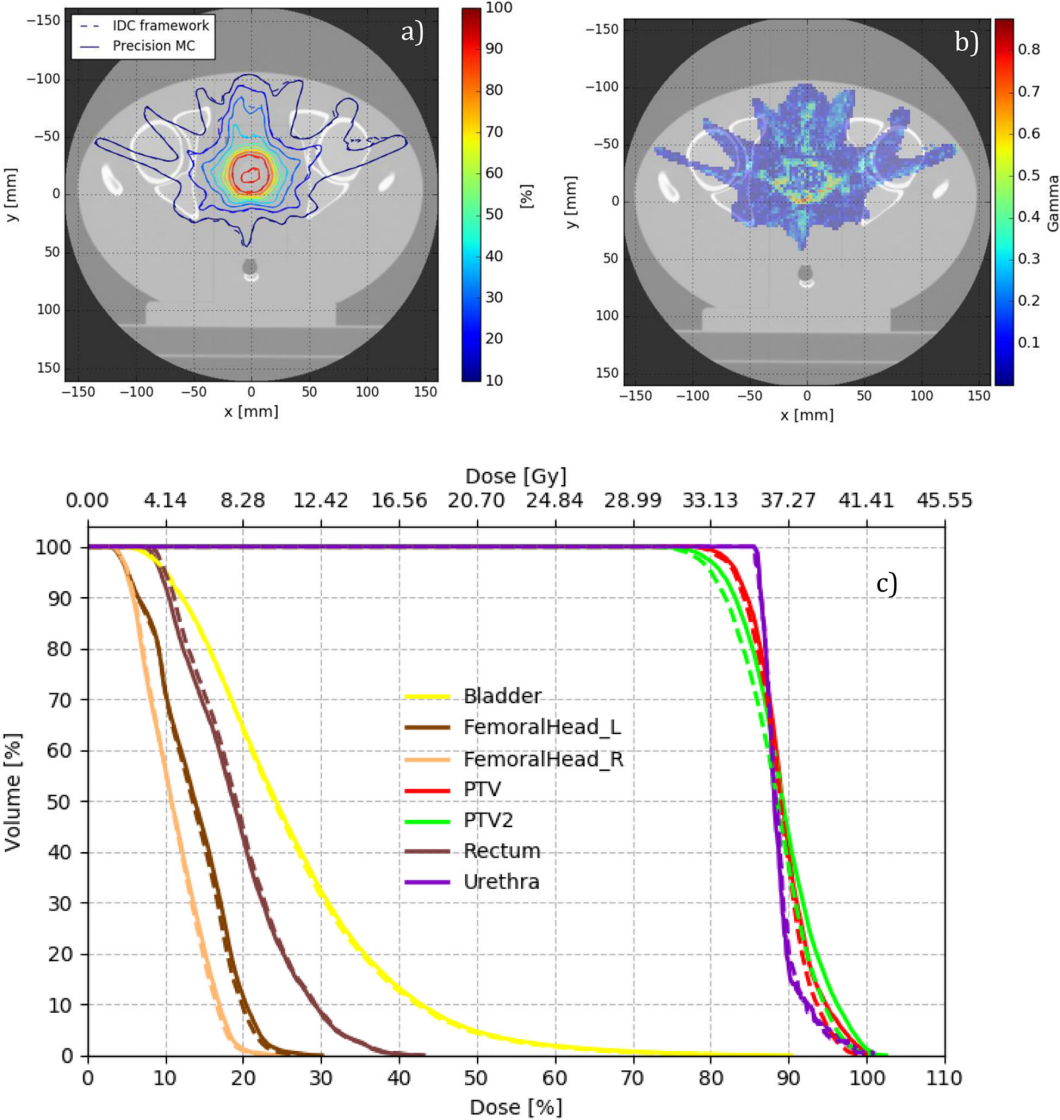
**a** Transversal slice with isodose plot, **b** gamma distribution, and **c** DVH for the prostate phantom case containing all MLC apertures (case 2). Precision MC solid, IDC dashed

For the two prostate phantom cases, comparative results between Precision MC and IDC are given in Fig. 4 (Only case 2 is shown for brevity, as it includes all MLC apertures while case 3 is a subset). While the gamma passing rates (99.9% for both cases) demonstrate generally an excellent agreement between the two methods, IDC shows slightly lower PTV mean doses (− 1.0% and − 0.8%, for case 2 and 3, respectively) as compared to Precision MC.

### Clinical cases

Due to the nature of this work to benchmark the Precision MC for clinical cases, different lung cases were investigated because it is expected that they are the most challenging cases with regard to accuracy of the dose calculation task. Table 4 shows results of dosimetric comparisons for all phantom and clinical cases. For the clinical lung cancer cases (cases 4–10), mean PTV doses agree within − 1.0 to 2.3%.

**Table 4:** Comparison of VOI doses and gamma passing rates for all ten cases (Case 1–3 are phantom cases, 4–10 are clinical cases). Gamma analysis performed using 2% (global) / 1 mm criteria with a 10% dose threshold

Case number	PTV mean dose IDC [Gy]	PTV mean dose Precision MC [Gy]	PTV mean dose change (IDC – Precision MC) / Precision MC [%]	Lung V20 IDC [%]	Lung V20 Precision MC [%]	Lung V20 change (IDC – Precision MC) / Precision MC [%]	Gamma passing rate [%]
1	39.1	39.1	0.0				96.2
1 FSPB		43.1	−9.3				36.2
2	36.8	37.1	−0.8				99.9
3	38.3	38.7	−1.0				99.9
4	56.4	55.6	1.4	11.9	11.7	1.5	99.6
5	54.0	54.2	−0.4	7.5	7.7	−1.5	98.1
6	58.7	58.7	0.0	2.9	2.9	0.0	99.7
7	56.4	55.1	2.3	1.0	1.0	0.0	99.4
8	66.9	67.6	−1.0	2.3	2.3	0.9	98.2
9	55.7	55.5	0.4	6.7	6.7	0.3	99.0
10	56.2	55.7	0.9	1.1	1.1	0.0	99.6

**Fig. 5:**
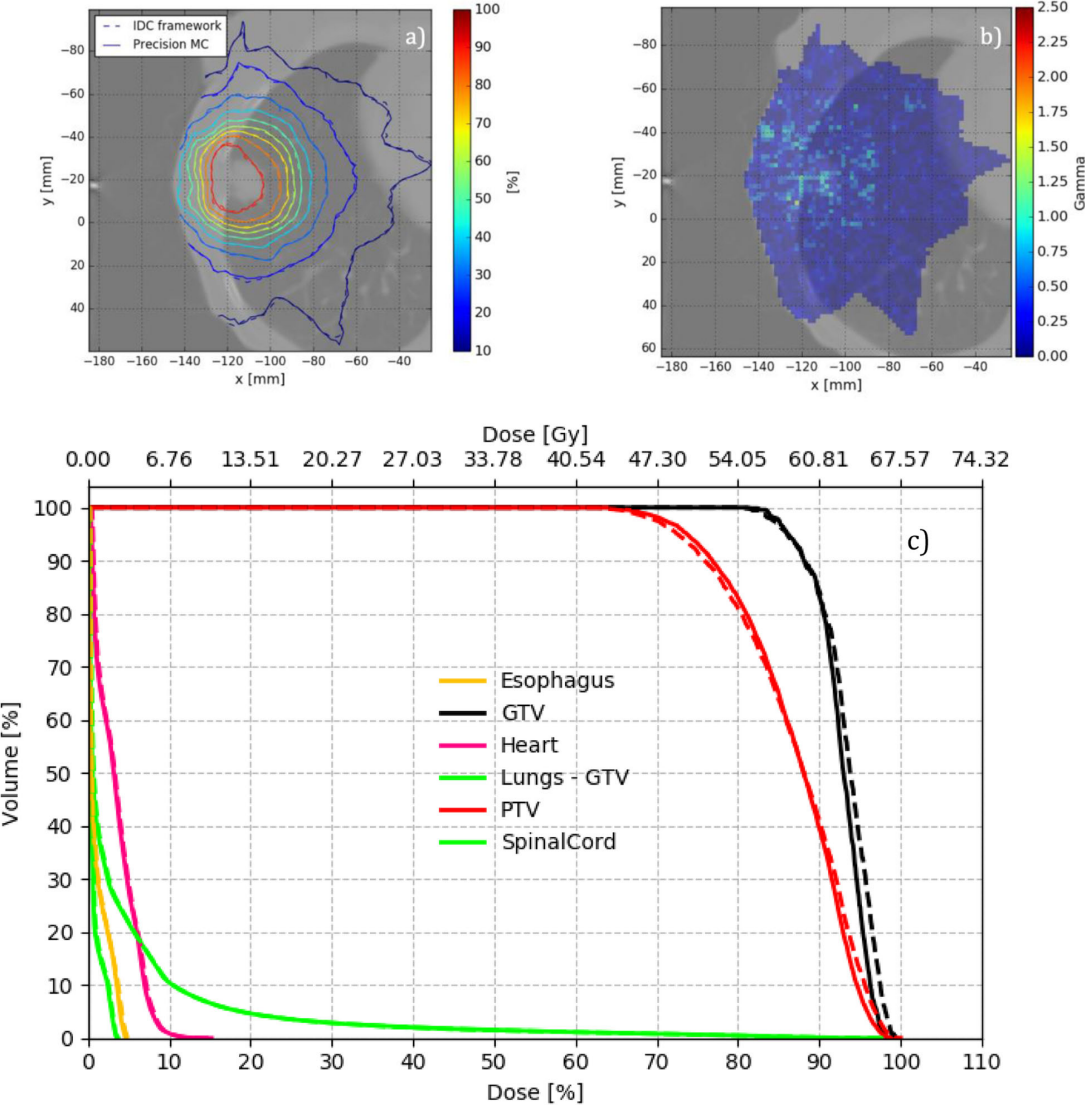
**a** Transversal slice with isodose plot, **b** gamma distribution, and **c** DVH for the clinical case 6 (lowest PTV mean dose change, “best-case”). Precision MC solid, IDC dashed

As an example, case 6 is considered in Fig. 5, where the isodose lines (a), the gamma distribution (b), and the DVH plots (c) are shown. In this case, mean PTV doses agree perfectly between Precision MC and IDC (best-case example).

**Fig. 6:**
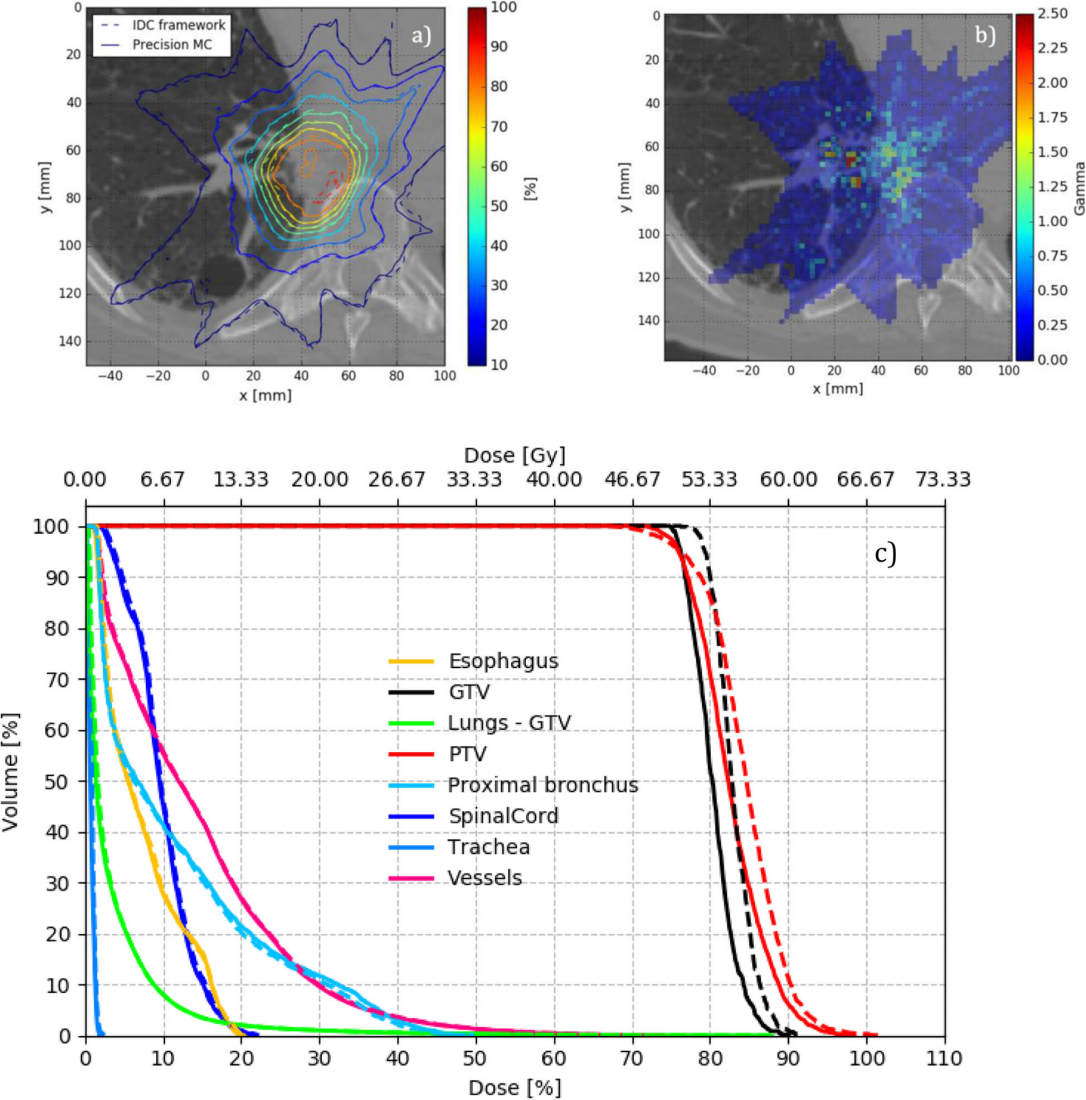
**a** Transversal slice with isodose plot, **b** gamma distribution, and **c** DVH for the clinical case 7 (highest PTV mean dose change, “worst-case”). Precision MC solid, IDC dashed

As a worst-case example, case 7 is considered in Fig. 6. For this case, PTV mean dose differs the most among all considered cases, as it is 2.3% higher for IDC compared to Precision MC.

In general, as can be seen from the previous figures, voxels failing gamma evaluation are primarily located at interfaces of tissues of different densities. On the other hand, lung V20 agrees between IDC and Precision MC within ±1.5% difference and gamma passing rates when comparing IDC to Precision MC are 98.1% or higher for all cases (as shown in Table 4). Calculation times for the seven clinical lung cases in Precision MC at native CT resolution and 2% requested uncertainty ranged between 2 min 11 s (case 6) and 8 min 7 s (case 5). If the axial plane resolution was reduced by a factor of two (offered in Precision MC as a “medium” resolution option) these reduced to 41 s and 2 min 11 s, respectively.

## Discussion

In this work, a commercially available dose calculation algorithm (Precision MC) for CyberKnife M6 treatments was benchmarked against measurements as well as against an independent dose calculation framework (IDC). A prior benchmarking study of the Precision MC for MLC included single beam tests in which Precision MC and FSPB calculations were compared to film and chamber measurements in a heterogeneous slab phantom [18]. That study showed good agreement (2%/1 mm 2D gamma passing rates of 91.2 ± 1.5%) between Precision MC and measurement in very low density lung substitute materials with localized anomalies due to a simplification in electron transport, which improved further after a modification was made to the electron transport algorithm (gamma passing rates of 96.6 ± 1.2%). That modification was introduced by the manufacturer before work for the current study started and is included in the Precision MC version used in our study. The work concentrated on MLC only, since MC-based methods are already existing for the other two collimator options (i.e. fixed size cones, IRIS) for the CyberKnife system. For this benchmark, different complexity levels were considered by looking at simple cases such as a homogeneous water tank, phantom cases reflecting lung or pelvis treatments, and clinical cases (all lung). By this means, it was possible to comprehensively investigate an entire spectrum of situations, which are not only of physics interest but also of clinical relevance.

Generally, for the cases considered, there is good agreement between Precision MC, IDC, and measurements. This was quantified by dose difference, distance to agreement, gamma evaluation, and DVH analyses (Tables 3 and 4).

The work also indicates the difficulty of accurately handling small fields in radiation therapy. Although IDC is based on the MC method – generally known as the most accurate dose calculation method – we observed dosimetric differences between IDC and measurements in the order of 4% for the smallest field. Since for the clinical cases, the treatment plans also include small fields, the observed differences for the clinical cases can partly be associated to this effect. Part of these differences can also be attributed to effects of partial source obscuration in the IDC MC model for the smallest field.

While for two prostate phantom treatment plans and seven clinical (lung) treatment plans the gamma evaluation between Precision MC and IDC results in passing rates > = 98.1%, we also showed the relevance of using MC instead of FSPB for heterogeneous anatomical situations. The addition of MC based dose calculation to MLC based CyberKnife treatments was thus important for treatments in regions such as lung, liver and mediastinal tumors.

Nevertheless, even though both algorithms – Precision MC and IDC – are based on the MC method, residual differences in the calculated dose distributions remain, which cannot be explained by statistical uncertainty only. Besides the different beam models used, the underlying MC transport methods differ (proprietary code and EGSnrc, respectively) and thus serve as an explanation for the observed differences. Moreover, as outlined in this work, both material conversion and electron track generation is handled differently in the two algorithms. Material conversion for photon interaction simulation on one hand, is simplified in Precision MC, assigning one of three materials (air, soft tissue or bone) to voxels instead of the 14 stoichiometric material compositions used in IDC. For electrons, on the other hand, interactions are pre-simulated in water. This leads to dose differences near inhomogeneous tissue (air/soft tissue/bone interfaces), which consequently are then observed in the corresponding phantom and clinical cases. As shown in Figs. 5b and 6b, Gamma values peak in regions of tissue interfaces (e.g. bronchi, pleura or the surface of the vertebral body) with IDC showing lower dose than Precision MC in air close to soft tissue and higher dose in soft tissue close to bone. These dose differences appear to be confined to small regions, suggesting the electron pre-simulation in water being the underlying cause. Even though peak differences in the dose of 2.5% were observed, these are generally acceptable differences for clinical routine work with gamma passing rates for 2% / 1 mm of 98.1% or greater [1].

Calculation efficiency of Precision MC is optimized (whereas in IDC it is deliberately not) and compares favorably to similar described frameworks [21]. This is to be expected, as IDC serves a “gold standard” purpose, not employing any efficiency improving approximations. While IDC takes 4–6 h to calculate dose to clinically acceptable mean statistical uncertainties of about 2% [11], Precision MC does so within 2.2–8.1 min at native CT resolution (note that if the resolution is reduced to 256 × 256 x number of slices, these times reduce to 41–132 s).

Both accuracy and efficiency of the Precision MC dose calculation are within clinically accepted limits rendering the system practical for routine use.

## Conclusions

Precision MC dose calculation for MLC based CyberKnife treatments was successfully benchmarked against IDC and measurements. Dose differences of Precision MC to gold standard IDC are sufficiently small for clinical use with greatly improved efficiency.

## Data Availability

The data that support the findings of this study are available from Accuray, Inc. but restrictions apply to the availability of these data, which were used under license for the current study, and so are not publicly available. Data are however available from the authors upon reasonable request and with permission of Accuray, Inc.
